# Letter from Adjunct Professor Martins, *of the Faculty of Medicine of Paris*

**Published:** 1839-07-06

**Authors:** 


### FOREIGN CORRESPONDENCE.

### LETTER FROM ADJUNCT PROFESSOR MARTINS, *of the Faculty of Medicine of Paris.*

### No. III.

*Observations upon the Radesyga, or Leprosy, of Norway.*

To the Editors of the Medical Examiner.

PARIS, *May 8th*, 1839.

THE medical journals have been lately filled with the discussion upon the effects of poisoning with the white oxide of arsenic, which has been going on between Messrs. Orfila and Bognetta. The disputants have become so violent upon the subject, that a long and cautious analysis has become necessary to discover where the truth lies: this I propose to attempt in my next letter. In the meanwhile, I shall occupy your attention with some researches of my own, the results of which have not yet been made public, but which it is my intention shortly to submit to the Academy of Medicine. They are upon the subject of the Radesyga, or Leprosy of Norway, which I had an opportunity of observing upon the spot, during the past year, and of having illustrated by some very capital drawings. Up to this time, we have been prevented from attaining an exact knowledge of this formidable affection, by two causes. The first of these is, that the Radesyga, limited as it is to a few provinces of Norway, has been observed only by the physicians of the country,—men, indeed, full of knowledge, but whose opportunities have not allowed them to study the other diseases of the skin, to be met with in the larger hospitals of Great Britain and France. And, on the other hand, so far as I am aware, no physician of the South of Europe, familiar with its cutaneous affections, has seen the Radesyga in the country in which it is endemic. This I was enabled to do for the first time at Drontheim, the capital of Northern Norway, the hospital of which contains a large number of lepers. Dr. Schultz, of that place, was kind enough to afford me every possible information respecting the patients, and even to act as interpreter in my interrogatories. I shall proceed to detail some cases observed, in order to bring before your readers the features of the disease.

*Case* 1.—John Olsen, twenty-four years of age, from a neighbouring village, a fisherman and labourer, not addicted to ardent spirits, born of parents never affected with the Radesyga, had himself been afflicted with it for above six years. His hair was sandy, skin rather brown. His nose, eyebrows, mouth, and skin, were covered with broad tubercles, often confluent, so as to form lobated tumours, through which runs a capillary reticulation, giving the mass a roseate hue. Three of these tumours were noticed at the root of the nose, and two others upon the alæ. None of them were ulcerated, except that above the left eyebrow. Some crusts were also observed upon the alæ nasi, and around the mouth, but moveable with the skin. The countenance of this man was truly frightful—his face resembled that of a lion. Upon the mucous portion of the lips were to be seen tubercles, smaller in size; the roof of the mouth, and inside of the cheeks, were irregularly studded with the same; there were none upon the tongue. The breast was disfigured with brown spots, and over its surface were scattered the cicatrices of old pustules. The arms were in the same condition; indeed, almost the general colour of the body was that of bistre; it was completely insensible. At the lower part of the forearm, and upon the hand, were observed indurated pustules, red at their base, which proves them to be the same suppurative tubercles so often remarked in secondary syphilis. The thighs and legs were of a reddish tint, the skin uneven, with tubercles scattered over its surface, some of which were several lines in diameter, had suppurated at the summit, and were covered with a crust. The inferior half of the legs was covered with scales. The penis, belly, and back, were sound. The appetite of the patient was good, although he was troubled with thirst; he suffered no pain in the skin, and his general health was not bad.

*Case* 2.—Mary Andersdatter, thirty-four years of age, a native of the village of Björnöer, was employed, before her illness, in tending sheep and cattle. Her attack dated as far back as the age of nineteen. Her parents were healthy. Hair, chesnut; face disfigured with the cicatrices of old ulcers; neck, breast, and back, of a light bistre colour, and covered with large, pruriginous papulæ. Upon the left arm these papulæ became actual tubercles, while the skin was thickened, dry, red, and cracked. The first phalanx of the thumb only remained, the two first phalanges of the index finger, and only the first of the three others. The stumps terminated in a surface rounded off, chapped, hard, and insensible to the touch, the skin completely covering the bone. On a line with all the articulations of the first phalanges with the metacarpal bones upon the palmar surface were deep fissures, hard, black, and secreting no pus. The whole forearm was insensible. On the right arm the thumb was entire, but bent inwards, tumefied, and very deeply chapped. The four fingers were reduced to a single phalanx; and two of them, the middle and ring fingers, presented stumps deeply chapped. At the elbows, the skin was tumefied, reddened, and indurated.

At the knees, below the patella, were enormous flakes of tumefied skin, chapped, rugous, uneven, covered with a crust of a yellowish-gray colour, like that which forms upon the proud-flesh of ulcers before it has been cauterized. The toes were reduced to little shapeless tubercles, scarcely projecting from the foot, and squeezed into one another, each having lost one or more phalanges. Upon only two of the toes did the nails remain. Upon the anterior half of the plantar portion of the left foot was a transverse ulcer, irregularly elliptic in shape, its bottom of a pale rose colour, and its edges perpendicular and oblique. An ulcer similar in character, but nearly cicatrized, was seen beneath the toe of the left foot.

Every physician acquainted with the subject of diseases of the skin, will not for an instant hesitate to recognise the nature of this disease, with the character of which I was at once impressed upon a first sight of the patients' countenances. This affection is no other than Tuberculous Leprosy, or the Elephantiasis of the Greeks; but the disarticulation of the fingers and toes, which is sometimes followed by that of the limbs in the affection which is met with in the colonies, a variety of the first, called by Robinson Elephantiasis anoestatos. These two affections, existing either in a blended or distinct form in the same individual, constitute the Radesyga. The victims of this loathsome disease often protract life for a considerable period, but they finally succumb to the attack, and Dr. Schultz informed me that tubercles were invariably discovered in their lungs.

The etiology of this horrible affection it is not easy to determine; and yet it is only from a knowledge of its causes that we can hope to arrive at the means of eradicating it. I shall proceed to mention the views of the physicians of the country upon the subject. The poor classes in Norway are in a condition of extreme wretchedness whenever the season is unfavourable, and the crops fail; they are compelled, in the interior, to live upon bread made from the bark of the birch tree, and, near the sea-coast, upon fish. How soon the appetite tires of this latter food, we all know; it becomes loathsome to a really extraordinary degree, and I believe that one would finally prefer to die of hunger than be confined to food of this description. In Norway, our whole party experienced this disgust for salmon—the first days of our sojourn, we found it delightful; but before very long, not an individual would touch it. But how does the miserable Norwegian attempt to revive his cloyed appetite? He buries the fish under ground, and allows incipient putrefaction to give it a flavour, before he eats it. It will readily be conceived what a fatal influence a prolonged nourishment of this kind must exercise upon the economy. In support of this opinion, a physician of Tromsol, Dr. Finch cited the following fact:—The Radesyga was unknown in a village not far from the town mentioned; a whale was cast ashore there, and the inhabitants of the village cut him up, and made their food of him for several months. A short time after, there appeared among them two cases of Radesyga, which I saw at Tromsol. This cause, however, is not the only one; for the disease is common in the district of Bergen, situated in the midst of mountains, the inhabitants of which are not at all ichthyophagous. We are, therefore, compelled, in this as in most other diseases, to forego a knowledge of its causes. But it is manifest that its extinction is to be hoped for only in an amelioration of the condition of the poorer classes, and in the application, on a large scale, of the laws of hygiene.

